# Soil Water Capacity, Pore Size Distribution, and CO_2_ Emission in Different Soil Tillage Systems and Straw Retention

**DOI:** 10.3390/plants11050614

**Published:** 2022-02-24

**Authors:** Vaida Steponavičienė, Vaclovas Bogužas, Aušra Sinkevičienė, Lina Skinulienė, Rimantas Vaisvalavičius, Alfredas Sinkevičius

**Affiliations:** Department of Agroecosystems and Soil Sciences, Vytautas Magnus University, K. Donelaičio Street 58, 44248 Kaunas, Lithuania; vaclovas.boguzas@vdu.lt (V.B.); ausra.sinkeviciene@vdu.lt (A.S.); lina.skinuliene@vdu.lt (L.S.); rimantas.vaisvalavicius@vdu.lt (R.V.); alfredas.sinkevicius@agrokoncernas.lt (A.S.)

**Keywords:** bulk density, soil pore structure, water retention, CO_2_ emission, long-term effect, no-tillage

## Abstract

The long-term implementation of crop rotation and tillage has an impact on the soil environment through inputs and soil disturbance, which in turn has an impact on soil quality. Tillage has a long-term impact on the agroecosystems. Since 1999, a long-term field experiment has been carried out at the Experimental Station of Vytautas Magnus University. The aim of this experiment is to investigate the effects of long-term various-intensity tillage and straw retention systems on soil physical properties. The results were obtained in 2013 and 2019 (spring rape was growing). According to the latest edition of the International Soil Classification System, the soil in the experimental field was classified as Endocalcaric Stagnosol (Aric, Drainic, Ruptic, and Amphisiltic). The treatments were arranged using a split-plot design. In a two-factor field experiment, the straw was removed from one part of the experimental field, and the entire straw yield was chopped and spread at harvest in the other part of the field (Factor A). There were three different tillage systems as a subplot (conventional deep ploughing, cover cropping with following shallow termination, and no-tillage) (Factor B). There were four replications. The long-term application of reduced tillage significantly increased soil water retention and improved the pore structure and CO_2_ emissions. Irrespective of the incorporation of straw, it was found that as the amount of water available to plants increases, CO_2_ emissions from the soil increase to some extent and then start to decrease. Simplified tillage and no-tillage in uncultivated soil reduce CO_2_ emissions by increasing the amount of water available to plants from 0.151 to 0.233 m^3^·m^−3^.

## 1. Introduction

A climate change project conducted by the European Environment Agency and the Centre for Environmental Research puts an emphasis on research into water retention in cultivated soils [1]. Research on the conservation of soil and its moisture resources is one of the most important topics in modern agronomy [2]. In the context of climate change, the efficient use of soil water is becoming one of the focal points of research on the productivity and stability of agroecosystems. Soil water retention depends directly on the structure of the soil pores, their size distribution, shape, continuity, tortuosity, and so on [3]. Soil pore structure depends on the soil texture, the content of organic matter as well as on crop and soil management practices and other factors affecting the aggregate structure of soil. Thus, soil water retention is influenced not only by the ratio of organic matter, sand, clay, and silt particles, but also by the chosen farming system, tillage intensity, and other factors [4]. Numerous researchers have found that no-tillage improves the soil structure, enabling better retention of soil moisture available to plants [5,6,7,8], and has more efficient usage [9] compared to conventional tillage. Many researchers agree that plant residues also play an important role in the retention of moisture in the soil [10].

Mechanical tillage affects the supply of nutrients to plants and all of the physical soil properties that are important for the soil moisture and air regime. These properties affect the formation of the biological potential of plants, and thus their yield. Plants have been found to suffer equally from both excessive soil looseness and excessive soil density. The effect of the density of individual soil layers on plants is uneven, as well. The soil density is also of varying importance at different stages of plant development. The density of the soil decreases when the soil is loosened, when the colloids swell from the moisture, and when the water in it expands during cooling. Plant roots and various microorganisms loosen the soil. Factors that increase and decrease soil density usually intertwine and interact within certain limits [11]. The effects of different tillage technologies on soil physical properties have been explored, and it has been found that soil bulk density was markedly higher in direct-drilling plots than in ploughed ones. No significant differences in soil bulk density were found among sustainable tillage technologies [12].

Soil consists of mineral particles and organic matter separated by pores filled with water or air. The content and the size distribution of pores depend on the size of the soil particles. The larger the pores, the fewer there are of them. Moreover, the wetter the soil, the more of its pores are filled with water. Due to meteorological climatic conditions, soil moisture levels vary from year to year [13]. In drought conditions, no-tillage technology allows for preserving higher soil moisture content at 0–10 cm. Therefore, it is considered a means of preserving soil moisture [14]. Soil air permeability depends mainly on large soil pores, total soil porosity, and the internal geometry of pores. Soil water retention is a key hydraulic property of soil, regulating soil functions and strongly influencing soil productivity [15].

Soil carbon dioxide (CO_2_) efflux is a physical process that is primarily driven by the CO_2_ concentration diffusion gradient between the upper soil layers and the atmosphere near the soil surface. Soil CO_2_ production is strongly influenced by environmental factors, including soil temperature, soil moisture, and macropores [16]. Soil pore characteristics are important for a large range of essential soil water and gas transport mechanisms as well as soil mechanical properties, such as soil friability [4]. One of the proposed measures to reduce CO_2_ emissions from the soil is the wider introduction and use of no-tillage farming in agricultural production [17]. Studies have shown that CO_2_ emissions from soil are proportional to the volume of mechanically compacted soil [18]. Tillage ensures soil inversion and the full incorporation of residues into the soil, while no-tillage (NT) is a management practice that eliminates tillage operations. There is no consensus on the effects of tillage practices on soil CO_2_ emissions in the literature. No-tillage and minimum tillage have been reported to reduce soil CO_2_ emissions compared to conventional ploughing (CP) in short-term tillage systems [19,20,21]. Another group of authors found higher soil CO_2_ emissions under NT than under CP and attributed them to the higher microbiological activity induced by crop residues on the soil surface and the relatively larger water-filled pore space under no-tillage [22].

The aim of this research is to investigate the effects of long-term various-intensity tillage and straw retention systems on soil hydrophysical properties and CO_2_ emissions. Our attention was focused on the evaluation of soil pore space distribution, soil pore size, volumetric water content, and the network of the macroporosity effects on the soil CO_2_ emission regime.

## 2. Results and Discussion

### 2.1. Soil Bulk Density

Soil bulk density is an indicator widely used to convert soil moisture content from weight-based measures into volume-based measures and to calculate soil porosity and void ratio. It fluctuates constantly throughout the year, and therefore is an unstable dimension. Soil bulk density is one of the main indicators characterising soil structure [23]. Research suggests that a too low soil bulk density leads to insufficient contact between soil and plant roots, while a too high bulk density results in the deterioration of aeration and increased soil penetration resistance, which inhibits root growth and development [24].

Experimental findings suggest that straw incorporation (S) at different soil layers (5–10 cm, 15–20 cm, and 30–35 cm) had no significant effect on soil bulk density (Table 1). A comparison of the studied tillage systems with conventional deep ploughing (CP) revealed no significant differences in soil bulk density at the 5–10 cm, 15–20 cm, and 30–35 cm soil layers. However, although insignificantly, soil bulk density differed among tillage systems in all soil layers (5–10 cm, 15–20 cm, and 30–35 cm). In addition, soil bulk density decreased in the reduced tillage and no-tillage treatments.

Similar trends were noted by scientists from the Lithuanian Research Centre for Agriculture and Forestry [25], who found that the soil bulk density varied in an optimal range from 1.28 to 1.36 Mg m^−3^ in reduced tillage plots, while the highest bulk density, between 1.35 and 1.41 Mg m^−3^, was found in deep ploughing plots. The determined similar trends after a long-term reduction in tillage in untilled soil had no significant effect on soil bulk density at 0–10 cm and 10–20 cm depths [26].

In summary, the addition of straw and the reduction in tillage intensity reduced soil density, as evidenced by our results.

### 2.2. Soil Pore Space Distribution

Soil type and land management are among the main factors that have an influence over the parameters of macropores in the soil [27]. The characteristics of soil macropores are important for a wide range of essential soil properties, such as friability [28].

**Figure 1:**
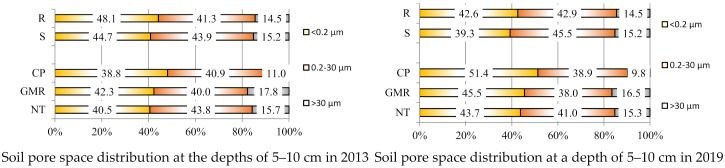

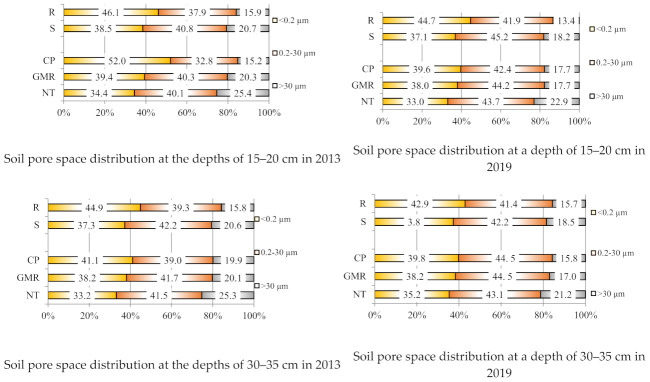
Soil pore space distribution (the content of micropores, mesopores, and macropores as a percentage of total porosity) at the depths of 5–10 cm, 15–20 cm, and 30–35 cm in 2013 and 2019. Notes: No significant differences at *p* > 0.05; Fisher LSD test vs. control. Other explanations are given in Table 1.

The findings obtained in 2013 (Figure 1) indicate that mesopores were the most prevalent in the upper soil layer (5–10 cm).

The plots with incorporated straw (S) had 2.61 percentage points (pp) larger mesopores than the plots without straw. A comparison of reduced tillage systems with conventional deep ploughing (CP) showed that there were more mesopores (2.90) in the upper soil layer (5–10 cm) in no-tillage (NT) treatments. Reduced tillage methods had insignificantly fewer mesopores compared to conventional deep ploughing (CP).

In the deeper 15–20 cm soil layer, the content of micropores was 7.61 pp lower in the plots with incorporated straw. The content of mesopores and macropores was also higher in these plots (2.82 pp and 4.78 pp, respectively). The highest content of micropores (52.02%) was found in the plots of conventional deep ploughing (CP). A higher content of mesopores (7.54 and 7.35 pp) was determined in the plots that were cover cropping with following shallow termination (GMR) and no-tillage (NT) plots. The lowest content of macropores (15.20%) was also found in conventional deep ploughing (CP).

The trends observed in 2013 in the deepest 30–35 cm soil layer were similar to those observed in the 15–20 cm soil layer. The plots with incorporated straw (S) had a lower content of micropores (by 7.61 pp) and higher contents of mesopores (by 2.82 pp) and macropores (by 4.78 pp). A comparison of different tillage systems revealed that the highest content of micropores (41.12%) was found in conventional deep ploughing (CP) plots. Higher contents of mesopores (2.71 and 2.52 pp) were found in all reduced tillage systems compared to conventional deep ploughing (CP). A higher content of macropores (0.25 and 5.42 pp) was found in the plots, cover cropping with following shallow termination (GMR) and no-tillage (NT) compared to conventional deep ploughing (CP).

The incorporation of straw (S) in 2019 increased the content of mesopores (by 2.61 pp) compared to the plots with removed straw (R). Comparison of reduced tillage systems with conventional deep ploughing (CP) showed that more mesopores (2.13 pp) were found in the upper (5–10 cm) soil layer when applying no-tillage (NT). Cover cropping with following shallow termination (GMR) reduced the content of mesopores, but it did so insignificantly when compared to conventional deep ploughing (CP).

A lower content of micropores (7.61 pp) was found in the deeper 15–20 cm soil layer in 2019 with incorporated straw (S), while the contents of mesopores and macropores were higher (by 3.30 pp and 4.78 pp, respectively). The application of different tillage systems revealed that the highest content of micropores (39.56%) was found in conventional deep ploughing (CP) plots. A higher content of mesopores (from 1.32 to 1.81%) was found in no-tillage (NT) plots, where cover cropping with following shallow termination (GMR) was applied.

A lower content of micropores (by 5.60 pp) was found in 2019 in the deepest 30–35 cm soil layer, where straw was incorporated (S); more mesopores (by 2.82 pp) and more macropores (by 4.78 pp) were found compared to the plots with removed straw (R). A comparison of different tillage systems showed that the highest content of micropores (39.77%) was found in conventional deep ploughing (CP) plots. More macropores (from 1.28 to 5.44 pp) were found in all reduced tillage systems compared to conventional deep ploughing (CP). The content of mesopores was unevenly distributed among different tillage systems.

It can be concluded that in 2013 and 2019, in the 5–10, 15–20, and 30–35 cm soil layers, in the plots where a straw was chopped and spread (S), the content of mesopores was higher and the content of micropores was lower, while that of macropores was higher compared to the plots with removed straw (R). Throughout the years analysed, more macropores were found in all the soil layers in the fields where straw was chopped and spread (S) compared to the plots with removed straw (R).

Using different tillage systems, the highest content of micropores was found in conventionally deep ploughed (CP) plots throughout the study years and in all the soil layers studied. No-tillage (NT) and cover cropping with following shallow termination (GMR) tended to increase the content of macropores compared to conventional no ploughing (CP). The content of mesopores in the soil was unevenly distributed under different tillage systems.

The content of mesopores in the soil was found to be higher in almost all of the plots where reduced tillage systems were used. Studies concerning the relationships between the macropore networks in the soil and plant roots under different land uses are of great interest, since the soil macropores affect the conductivity of water, air, and mineral solutions in the soil. The macropores of the soil are large soil voids. Plant roots use them as paths for growth. The wormholes, soil cracks, and voids between aggregates are often associated with a high degree of variability in the transport of the gases, moisture [29], and dissolved substances through the soil [30,31].

Many studies have shown that soil porosity and soil infiltration can be induced by plant roots [32,33]. They found that macropores in the soil formed by plant roots are a major factor affecting downward water movement in pastures. A recent study revealed that the complexity of macropore networks may be linked with the age of the soil in the pedogenesis process [34].

### 2.3. Soil Water Retention Capacity

**Figure 2:**
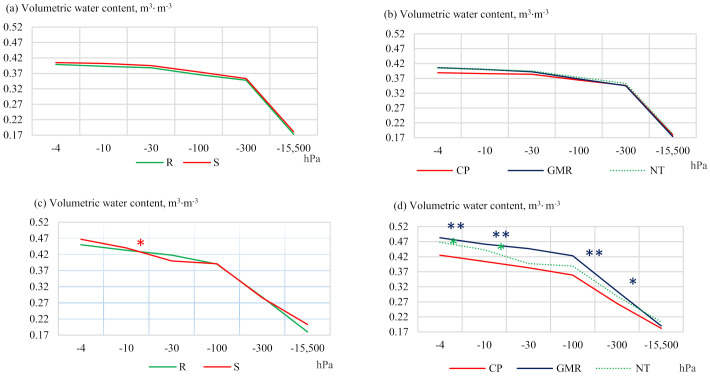
Soil water retention capacity at a depth of 5–10 cm in a (**a**,**b**) 2013 and (**c**,**d**) 2019. Notes: Significant differences at * *p* ≤ 0.05 > 0.01; ** *p* ≤ 0.01 > 0.001; Fisher LSD test vs. control. Other explanations are given in Table 1.

Water retention in the soil and the supply of moisture to plants during their growing season are relevant issues in both dry and temperate climates [35]. Soil fertility is determined not only by water and nutrient regimes in it, but also by soil type and texture, as well as the tillage technologies applied. The interest in sustainable crop production systems has been growing in Lithuania. They not only simplify tillage, save time and energy resources, and reduce emissions, but also affect the physical processes of the soil. The application of reduced tillage methods changes the soil bulk density, the total and air-filled porosity, as well as its chemical properties. An investigation of soil water retention capacity carried out in 2013 showed that the soil from the plots where straw was chopped and spread (S) contained from 1.4 to 4.6% more moisture in the 5–10 cm layer compared to the plots with removed straw (R) (Figure 2). The soil of no-tillage (NT) plots had from 1.7 to 3.6% higher moisture content at different pressure levels compared to conventional deep ploughing (CP). Plant residues remaining on the soil surface were found to reduce evaporation, leaving more water in the soil [36]. An analysis of the data of soil water retention capacity from 2019 revealed that in the treatment with chopped and spread straw (S) (Figure 2), the moisture content was evenly distributed among the soil layers, compared to the plots with removed straw (R). In the upper (5–10 cm) soil layer, significantly (1.6%) higher soil moisture content was found at a pressure of −10 hPa in the soil with chopped and spread straw (S).

**Figure 3:**
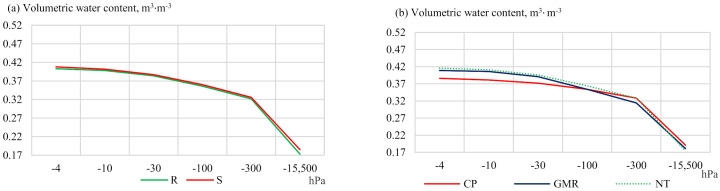

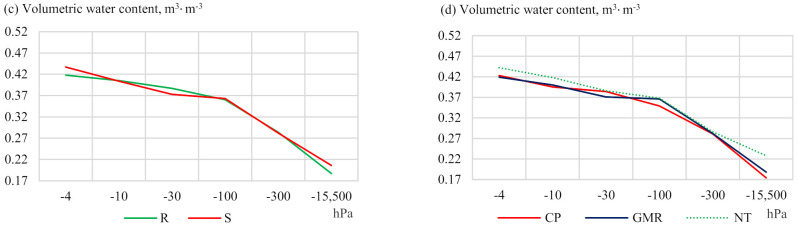
Soil water retention capacity at a depth of 15–20 cm in (**a**,**b**) 2013 and (**c**,**d**) 2019. Notes: No significant differences at *p* > 0.05; Fisher LSD test vs. control. Other explanations are given in Table 1.

The moisture content in the 15–20 cm layer in 2013 was found to be 0.003–0.012 m^3^ m^−3^ higher in the soil where the straw was chopped and spread (S) compared to the plots where the straw had been removed (Figure 3).

The trends observed in the 15–20 cm soil layer in 2013 were similar to those in the 5–10 cm soil layer. At different pressure levels up to −15,500 hPa, soil moisture was best preserved in the treatments of no-tillage (NT) compared to conventional deep ploughing (CP) (Figure 3). The moisture content in 2019 under no-tillage in uncultivated soil (NT) and cover cropping with following shallow termination (GMR) was from 3.7 to 17.8% higher compared to that for conventional deep ploughing (CP) at different pressures. The insertion and spreading of straw had different effects on soil moisture retention at different pressures (Figure 3).

**Figure 4:**
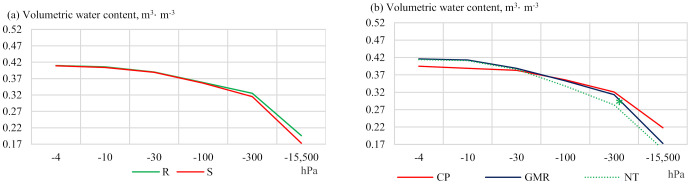

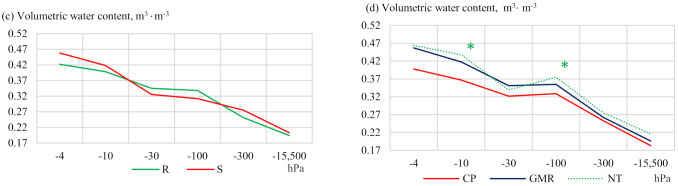
Soil water retention capacity at a depth of 30–35 cm in (**a**,**b**) 2013 and (**c**,**d**) 2019. Notes: Significant differences at * *p* ≤ 0.05 > 0.01; Fisher LSD test vs. control. Other explanations are given in Table 1.

The analysis of the values of soil water retention capacity in the 30–35 cm layer in 2013 and 2019 shows that the insertion and spreading of straw had different effects on soil moisture at different pressures (Figure 4). Compared to the conventional deep ploughing (CP) in 2013, other reduced tillage methods exhibited better soil water retention capacity at −4 to −30 hPa. As the pressure level increased, the soil moisture content decreased compared to conventional deep ploughing (CP). A significant decrease in soil moisture content (11.5%) was observed at −300 hPa in the no-tillage (NT) treatment compared to conventional deep ploughing (CP).

After analysing the results obtained in 2019, very similar tendencies were found in the deepest soil layer (30–35 cm) and in the upper (5–10 cm) soil layer. In the 30–35 cm soil layer (Figure 4), 3.6 to 18.0% higher moisture content was found in the plots with no-tillage (NT) and shallow green manure rotovating compared to conventional deep ploughing (CP). A significantly higher soil moisture content was determined using −10 and −100 hPa pressures in the no-tillage plots (NT) (19.3% and 14.0%, respectively) compared to conventional deep ploughing (CP).

For farmland soils, soil water retention varied considerably with tillage practices in space and time [37] because of the changes in pore-size distribution. The pore-size distribution of a soil defines its water retention and transfer behaviours (i.e., drainage, retention, and storage) [38]. Tillage-induced fluctuations in pore size distribution alter the characteristics of the distribution of functional pore groups. Conventional deep tillage (CT) practices such as ploughing and subsoiling rearrange soil particles physically, break the connective pores, and change pore size distributions, which directly alters the soil water retention capacity [39].

Summarising our research results for all of the studied years allows us to conclude that the moisture content in the soil with chopped and spread straw (S) was distributed unevenly. The water capacity data from 2013 revealed that the soil that had the straw chopped and spread (S) had a higher moisture content in the upper (5–10 cm and 15–20 cm) soil layers compared to the plots with the straw removed (R). In 2013 and 2019, a higher moisture content was found at different pressures in the upper (5–10 cm) soil layer, in no-tillage (NT) plots compared to conventional deep ploughing (CP). Throughout the years analysed, in the deeper soil layers (30–35 cm) at a pressure of −30 hPa, in the plots in which no-tillage (NT) or cover cropping with following shallow termination (GMR) were applied, the soil moisture content was higher than in conventionally deep ploughed (CP) plots. The results are very similar in the no-tillage (NT) system, where soil disturbance is minimal and the surface is continuously covered with crop residues, which supports the formation of a well-developed pore system with higher water retention capacity and more efficient pore connectivity than a tilled soil [40]. However, continuous NT may, in some cases, increase the risks of long-term soil compaction [41], as it was found that the air-filled porosity increased, but only in the first year after tillage. When a 9-year NT system was tilled, soil water retention at both field capacity (−330 hPa) and permanent wilting point (−15,000 hPa) was decreased [42].

Tillage causes changes in the soil pore system, affecting processes related to air and water fluxes and mechanical resistance to root growth. Conventional tillage generally promotes whole soil disturbance up to the 0–25 cm depth, which leads to soil, water, nutrient, and organic carbon losses due to erosion, organic carbon losses through faster mineralisation, and subsurface soil compaction [43].

The results show that a higher moisture content was found in the 5–10 cm soil layer in the fields where no-tillage was applied to uncultivated soil at different pressures. Covering the fields with straw in the top layer of the studied soil resulted in higher soil moisture than after removing the straw.

### 2.4. CO_2_ Emission and Volumetric Water Content Available to Plants

CO_2_ emissions are quite inconsistent; some authors found similar CO_2_ emissions from no-tillage in uncultivated soils, sustainable, and conventional tillage [44], while others found higher emissions using no-tillage technology or argued that CO_2_ emissions from no-tillage into uncultivated soil are higher only in some periods and lower in others [45]. There are also some researchers who claim that CO_2_ emissions from no-tillage are significantly lower only in the short term after tillage [46].

Various studies have shown that factors such as soil structure, temperature, humidity, pH, the carbon content in stable and unstable soil organic matter, and nitrogen content in the soil contribute to soil CO_2_ emissions [47]. We determined the dependence of CO_2_ emissions from the soil on plant species and growth stage but did not observe a significant effect of tillage technologies on CO_2_ emissions [48]. The data we obtained confirm the research results of other scientists on different crops (spring rape in 2013 and 2019).

The results of the experiments carried out in 2013 and 2019 (Table 2) show that straw incorporation (S) did not have a significant effect on CO_2_ emissions from the soil under different tillage systems compared to conventional ploughing (CP). In 2019, the addition of straw reduced CO_2_ emissions and increased the volumetric water content available for the plants compared to conventional tillage.

The sensitivity of CO_2_ emissions to water content and temperature increases after tillage. The impact of tillage on CO_2_ emissions from the soil is expected to last for about 35 days; we observed that CO_2_ emissions increase if the soil is moist, and the emissions are very high after heavy rains [49]. The results obtained are in agreement with the results indicating that CO_2_ emissions were 1.3% and 2.0% lower in the places where plant residues were spread (S) compared to straw removal. Many researchers have concluded that soil water content is considered to be the most influential environmental factor controlling soil surface CO_2_ efflux [50,51]. Moreover, water content, when treated sustainably without abrupt soil disturbance, is higher than under conventional tillage (CT) [52].

Mechanical tillage affects all the physical soil properties that are important for the soil moisture and air regime. One of the possible solutions to water scarcity in the soil is the application of sustainable tillage systems. Not only the choice of tillage method determines the soil moisture, but also precipitation and air temperature (heat content) determine the soil moisture content and the growth conditions of plants [53]. In the vegetation period of plants in 2013 and 2019, precipitation was lower than the long-term average. However, the water available to plants was determined by applying simplified tillage systems compared to conventional ploughing. Researchers argue that sustainable agricultural technologies reduce the negative impact on the environment and greenhouse gas CO_2_ emissions from the soil [54]. Our results show that the intensity of CO_2_ emissions was lower in the fields where cover cropping with following shallow termination (GMR), while in the fields no-till trends than in deep ploughing were found. Most authors argue that soil temperature is associated with CO_2_ and NO_2_ emissions: as the soil temperature increases, so does the release of these gases into the atmosphere. Thus, this effect becomes a positive feedback loop in the climate system [55]. Our results confirm that meteorological conditions are important (Tables 4 and 5); precipitation decreased during the vegetation period and active temperatures were also lower compared to the long-term averages. For these reasons, not only the way the soil is cultivated but also the meteorological conditions have an impact on CO_2_ emissions from the soil.

Irrespective of the incorporation of straw, it has been found that as the amount of water available to plants increases, CO_2_ emissions from the soil increase to some extent and then start to decrease. The cubic equation shows the relationship between CO_2_ emissions and the water available to plants: Y = 26,460x^3^ − 14,737x^2^ + 2712.3x − 160.95R^2^ = 0.547.

Deep ploughing increases CO_2_ emissions from the soil as the amount of water available to plants increases from 0.142 to 0.184 m^3^ m^−3^. A linear positive moderately strong relationship was determined (R^2^ = 0.395). Simplified tillage and no-tillage into uncultivated soil reduce CO_2_ emissions by increasing the amount of water available to plants from 0.151 to 0.233 m^3^ m^−3^. A linear strong negative reliable dependence was determined (R^2^ = 0.843 **).

## 3. Material and Methods

### 3.1. Site Description

A long-term field experiment was established in 1999, at the Experimental Station of Vytautas Magnus University (54°52′50″ N latitude and 23°49′41′′ E longitude). The results of the first decade from the start of the experiment are presented in the publication [56]. In this publication, the results following the years 2013 and 2019 are presented (spring rape was growing). According to the international soil classification system [57], the soil in the experimental field was classified as Endocalcaric Stagnosol (Aric, Drainic, Ruptic, and Amphisiltic). The texture of the topsoil is loam, containing a medium content of plant-available phosphorous and potassium (Table 3). The long-term experiment was laid out in a split-plot design with four replications and a total of 24 plots. The initial size of the plot was 102 m^2^ (6 m × 17 m) and the size of the harvested plot was 30 m^2^ (15 m × 2 m).

### 3.2. Experiment Design and Agricultural Practices

In the agroecosystem crop rotation of spring oilseed rape (*Brassica napus* L.), winter wheat (*Triticum aestivum* L.) and spring barley (*Hordeum vulgare* L.), the most popular crops grown in Lithuania, were chosen. In a two-factor field experiment, the straw (Factor A) spring barley was removed (R) from one part of the experimental field, and in the other part of the field, the entire straw yield was chopped and spread (S) at harvest. The three different tillage systems (Factor B) were investigated as subplots: (1) conventional deep ploughing (CP) in autumn at a depth of 23–25 cm, (2) cover cropping with following shallow termination before the next crop sowing (GMR) at a depth of 5–6 cm, and (3) no-tillage (NT). All the tillage systems were tested in both halves of the experiment with and without the straw. After harvesting, the plots of conventional ploughing were cultivated with disc implements and deep ploughing in autumn. White mustard (*Sinapis alba* L.) as a cover crop for green manure on stubble was sown only in GMR plots right after the harvest of winter wheat and spring barley. The plots with no-tillage were neither tilled in autumn nor in spring. The crops were sown with a Väderstad Rapid 300C Super XL (Väderstad, Sweden) drill without surface cultivation with discs.

### 3.3. Meteorological Conditions

In 2013, during the growing season (Table 4), the average monthly temperatures were lower than the long-term averages. Precipitation was unevenly distributed during this period. In the vegetation period of 2019 (Table 5), the average monthly temperatures were very similar to the long-term averages, but the precipitation decreased less during the whole vegetation period than the long-term average.

Comparing the sums of the three-year active temperatures, it was found that they were the highest in 2019. In all the years studied, the precipitation was lower than the sum of the long-term precipitation averages.

In summary, the climatic parameters of the three-year vegetation periods differed from each other and from the long-term average conditions (since 1974). The meteorological conditions are closely related to the agrophysical and hydrophysical properties of the soil.

### 3.4. Methods and Analysis

The soil sampling was performed in 2013 and 2019 after the sowing of spring rape. Undisturbed core samples were collected using stainless steel rings (100 cm^3^ volume) from depths of 5–10, 15–20, and 30–35 cm to determine the soil water release characteristics (hPa) in six replications. The characteristics of water release were determined at −4, −10, −30, and −100 hPa (in a sandbox) and at −300 hPa (in a 15 bar pressure plate extractor). Loose soil samples were used to determine the water content at −15,500 hPa tensions by using a high-pressure membrane apparatus [58]. The water content at −100 and −15,500 hPa was considered as field capacity (prevailing in Europe) and as a permanent wilting point, respectively. The soil pore space distribution of the contents was performed of micropores <0.2 µm, mesopores 0.2–30 µm, and macropores >30 μm as a percentage of total porosity at the depths of 5–10 cm, 15–20 cm, and 30–35 cm. The water content between these two suctions was identified as the plant’s available moisture content. Soil pore space distribution and soil water retention capacity were calculated from the data collected [59,60].

Soil dry bulk density in the undisturbed monolith samples was determined using stainless steel rings (volume of the monolith–100 cm^3^) from the middle of each profile. The samples were dried in an oven at 105 °C for 48 h.

Soil CO_2_ emissions were measured using the Infra-Red Gas Analyser. A portable, automated soil gas flux system LI-8100A with a 8100-103 chamber, analyser LI-8100A (LI-COR Inc., Lincoln, NE, USA) was used for the measurements of the soil surface CO_2_ efflux (μmol m^−2^ s^−1^). In each record plot, in spring, rings 20 cm in diameter were installed in the soil, and three measurements were made in each plot [61]. CO_2_ efflux was carried out three times per growing season, at the same time of the day (from 10 a.m. to 5 p.m.) and at fixed locations in the field.

All experimental data were processed using two-factor analysis of variance (ANOVA) from the statistical software package SYSTAT, version 10 [62]. The significance of differences among the treatments was estimated by the least significant difference (LSD) test. If there was a significant difference between a specific treatment and the control treatment, its probability level was indicated as follows: * when *p* ≤ 0.050 > 0.010 (significant at 95% probability level), ** when *p* ≤ 0.010 > 0.001 (significant at 99% probability level), *** when *p* ≤ 0.001 (significant at 99.99% probability level).

The method of correlation regression analysis was applied to evaluate the causality of the studied traits. We used the program STAT ENG from the package ANOVA [63,64].

## 4. Conclusions

After 20 years, the tillage system’s application and straw retention did not have a significant effect on soil bulk density. Soil bulk density ranged from 1.47 to 1.58 Mg m^−3^. Compared to deep ploughing, neither cover cropping with subsequent shallow termination nor no-tillage increased soil bulk density. Reduced tillage did not adversely affect the soil pore space distribution, as in many cases it was greater than under deep ploughing. No-tillage and cover cropping with subsequent shallow termination increased the content of macropores compared to conventional ploughing. The mesopores in the soil were unevenly distributed under different tillage systems. The moisture content in the soil with chopped and spread straw was unevenly distributed. Water content data from 2013 revealed that the soil with straw had a higher moisture content in the upper (5–10 cm and 15–20 cm) layers compared to the plots with the straw removed. In 2013 and 2019, at different pressures, a higher moisture content was found in the upper (5–10 cm) soil layer in no-tillage compared to conventionally ploughed plots. Regular straw retention and cover cropping with following shallow termination and no-tillage increased the CO_2_ efflux in proportion to soil tillage intensity.

## Figures and Tables

**Table 1:** The influence of tillage intensity and straw retention on soil bulk density (Mg m^−3^) at different soil depths.

Soil Depths, cm	Factor A		Factor B		
	R	S	CP	GMR	NT
5–10	1.52	1.51	1.57	1.52	1.53
15–20	1.53	1.50	1.53	1.53	1.49
30–35	1.52	1.48	1.56	1.47	1.47

Notes: No significant differences at *p* > 0.05; Fisher LSD test vs. control. Factor A: R—straw removed (control), S—straw chopped and spread. Factor B: CP—conventional deep ploughing (control), GMR—cover cropping for green manure with following shallow termination before next crop sowing, NT—no-tillage.

**Table 2:** The influence of tillage intensity and straw retention on CO_2_ emissions from the soil surface (0–10 cm) and volumetric water content available to plants.

Indices	Year	Factor A		Factor B		
		R	S	CP	GMR	NT
CO_2_ emission, μmol s^−1^	2013	3.93	3.85	3.95	3.90	3.95
	2019	3.73	3.68	3.67	3.47	3.68
Volumetric water content, m^3^ m^−3^	2013	0.193	0.194	0.184	0.194	0.191
	2019	0.210	0.188	0.177	0.233	0.186

Notes: No significant differences at *p* > 0.05; Fisher LSD test vs. control. Other explanations are given in Table 1.

**Table 3:** Experimental plot soil characteristics (0–25 cm).

Index	Average Value
Sand %	35.6
Clay %	19.0
Silt %	45.4
pH_KCl_	7.7
Soil organic carbon (SOC) g kg^−1^	16.6
Available phosphorus (P_2_O_5_) mg kg^−1^	116.0
Available potassium (K_2_O) mg kg^−1^	111.0

**Table 4:** Average temperature (°C) and the sum of active temperatures (SAT) during the growing season in 2013 and 2019, Kaunas Meteorological Station.

Year/Month	04	05	06	07	08	SAT
2013	6.1	12.3	15.6	17.6	16.6	1675.6
2019	9.1	13.0	19.8	17.1	18.1	1800.2
Long-term average 1974–2019	6.9	13.2	16.1	18.7	17.3	1918.5

SAT = sum of active temperatures (≥10 °C).

**Table 5:** Precipitation (mm) during the growing season in 2013 and 2019, Kaunas Meteorological Station.

Year/Month	04	05	06	07	08	Sum
2013	56.5	63.8	45.9	118.5	67.2	351.9
2019	0.6	29.9	49.4	60.1	68.2	208.2
Long-term average 1974–2019	41.3	61.7	76.9	96.6	88.9	365.4

## Data Availability

Not applicable.
